# The relationship between leadership and adaptive performance: A systematic review and meta-analysis

**DOI:** 10.1371/journal.pone.0304720

**Published:** 2024-10-18

**Authors:** Alice Bonini, Chiara Panari, Luca Caricati, Marco Giovanni Mariani

**Affiliations:** 1 Department of Economics and Management, University of Parma, Parma, Emilia-Romagna, Italy; 2 Department of Humanities, Social Sciences and Cultural Industries, University of Parma, Parma, Emilia-Romagna, Italy; 3 Department of Psychology, University of Bologna, Bologna, Emilia-Romagna, Italy; COMSATS University Islamabad - Wah Campus, PAKISTAN

## Abstract

This research presents a comprehensive review and meta-analysis of literature to examine the impact of various leadership styles on organizational adaptive performance (AP). AP is essential for job performance, especially in environments undergoing rapid changes. Previous reviews on AP found that transformational and self-leadership had had a positive influence on job adaptivity, while the relationship between other leadership styles and AP had not been clear. First, authors outlined the theoretical framework of AP and leadership, clarifying how job adaptivity and the different leadership styles are defined and discussed in the scientific literature. Subsequently four scientific databases were explored to identify studies that investigate the Leadership and AP’ relationship. 32 scientific articles and 2 conference papers were investigated for review, of which 31 were used to conduct a meta-analysis; 52 different effect sizes from 32 samples were identified for a total sample size of 11.640 people. Qualitative synthesis revealed that the influence of different leadership styles on AP depended on contextual variables and on aspects related to the nature of the work. Moreover, it was found that leadership supported AP through motivational and relational aspects. Through this meta-analysis, it was found that a significant positive relationship between leadership and AP existed (*Z*r = .39, SE = .04, p < .001. 95%CI [.32, .47], r = .37). However, no differences emerged from the different leadership styles examined in the studies. This review deepens the importance of leadership as organizational factor that affect the employees’ likelihood of dealing with continuously emergent changes at work, extended the search to emerging leadership approaches to highlight the value of collective contributions, ethics, and moral and sustainable elements that could positively affect AP.

## Introduction

In order to remain competitive on the labor market, companies are increasingly requesting their human resources to be able to adapt to changes and to learn new skills. For instance, technology applied to work has been constantly evolving and requires lifelong learning efforts in the acquisition of new digital skills functional to its use [1,2]. Most of the scientific literature is centered around individual differences, rather than around organizational factors that can affect job adaptivity [3]. Reflecting on contextual aspects, it is possible to read leadership as an organizational resource that activates motivational processes promoting high performance, commitment, and proactive behaviors [4].

Particularly, leadership plays a crucial role in involving the worker in proactive and positive attitudes in facing change and promoting adaptive performance (AP), by way of modifying their organizational features and encouraging bottom-up initiatives, such as job crafting [5–7] This suggests that leadership, focused on human resources by encouraging followers’ self-determination and developing their intrinsic motivation, creates the ground to foster adaptivity [8]. Individual or group adaptation passed through the leader’s ability to reinforce collaborators’ personal skills, such as tension to results and autonomy, and the leader’s capacity to pay attention to his/her followers’ individual motivational differences and needs. Following the self-determination theory [9], transformation in collaborators occur when, the leadership contribute to satisfied their basic human psychological needs (autonomy, competence and relatedness) are satisfied. In the same way, paying attention to relational dynamics helped create and maintain trust in leaders and stimulated adaptive performance by sharing and managing emotional states related to changes [10].

Despite the recognized importance of leadership in facilitating adaptive performance, the understanding of how different leadership styles specifically contribute to this dynamic remains fragmented in literature. An updated systematic review and meta-analysis are necessary to consolidate existing research, identify gaps in our knowledge, and understand the nuanced ways in which leadership can effectively foster an environment conducive to adaptability. This will enable organizations to develop more targeted strategies in leadership development, directly addressing the evolving challenges of the modern workplace.

Based on these assumptions, the primary goal of this review was to emphasize how the relationship between adaptivity to work changes and leadership had been studied as an organizational antecedent that could promote or inhibit one’s adaptive job performance. Particularly, the purpose of this study was to provide a contribution to the existing literature on adaptive performance, by conducting a systematic review and a meta-analysis that would allow for a qualitative and quantitative synthesis of the scientific evidence currently available on the relationship between leadership and AP.

The secondary aim of this review was to dig deeper into the theoretical distinction among different leadership styles, so to understand what peculiarities, differences and dimensions characterize the different styles that could potentially influence AP. So, to guide this exploration, we pose three research questions:

RQ 1. How does leadership influence an individual’s adaptive performance?
H1: We hypothesized a strong and positive relationship between leadership and Adaptive Performance.
RQ2: What specific leadership styles are most effective in promoting adaptive performance among employees?
H2: We hypothesized a different level of strength in the relationship between styles and Adaptive Performance; in particular, we hypothesized that leadership styles emphasizing members’ involvement, such as transformational leadership, emergent approaches, and members’ leadership, would be more strongly related to Adaptive Performance than control-based leadership approaches, such as transactional or directive ones (H3).

With these aims, the following sections will detail the theoretical foundations of AP and leadership, leading into a comprehensive discussion based on the systematic review and meta-analysis that synthesizes our findings on the interplay between leadership, with his styles, and AP.

We assumed that the findings could be able to help organizations understand what leadership-related strategies and tools to use to decrease change resistance and promote adaptivity.

### Literature background

#### Adaptive performance: Definition and antecedents

The construct of AP, coined by Neal and Hesketh [11], was born to differentiate between task and contextual job performance [12] with reference to a set of behaviors that arising from a person’s ability to transfer his/her own knowledge to different contexts and to adapt to new job requirements (Allworth and Hesketh, 1999 [13]), but nowadays, it may be assumed that both the task and contextual job performance can be declined in an adaptive way [14]. Park and Park, while studying AP-related literature found that some construct definitions emphasized personal characteristics, while others focused on behavioral responses or on cognitive aspects of knowledge acquisition and skills transfer [5,15]. Despite these differences, all definitions considered adaptation as the implementation of behaviors in response to changing working conditions. Pulakos, in particular, proposed a multidimensional model of adaptive performance based on directly observable and measurable behaviors identifying eight dimensions, which involved task and contextual characteristics, connected with: one’s ability to deal with unpredictable and stressful work situations, managing frustration through resilience and directing one’s efforts towards functional solutions; one’s capacity to perform dynamically, taking actions in mutable situations by changing goals, priorities or actions; learning and acquiring new tasks, procedures or working methods by using past experiences to anticipate possible changes and, finally, to be creative in coping with new situations or to find new work resources and be able to adapt—cognitively, emotionally and physically—in interpersonal relationships, as well as in heterogeneous cultural and social environments [16,17]. All these assumptions implied that adaptive performance should be seen as a form of proactive adaptation that implies a degree of event anticipation as an effective response to change [11,18]. For instance, the new model of work role performance proposed by Griffin, Neal and Parker was thought of as innovative, because it was multidimensional and structured starting from insecurity and uncertainty in the work environment. The authors incorporated proficiency, adaptivity and proactivity into three different levels (individual, group and organizational), as key elements of the response to changes. This model introduced the concept of adaptivity, both individually and collectively, with reference to the degree and the way in which people cope with and support organizational changes, either individually or as members of a group and organization [19].

Many studies investigated the personal features that could influence adaptivity, whereas contextual, situational and organizational aspects remained little explored [13,20,21] In this sense, the systematic review of Park and Park [15] was one of a few studies that, in addition to highlighting individual antecedents, examined contextual and organizational AP antecedents, by emphasizing the crucial role of leadership, analyzed both at the organizational and individual levels. The authors reported that transformational leadership had an impact at the collective level, as it contributed to creating a cooperative and sharing climate that allowed for openness when solving problems in non-traditional ways and that provided the motivation for employees to make an extra effort when coping with complexity [22,23]. At the individual level, the authors focused on self-leadership affecting individual adaptivity at the cognitive, behavioral and emotional levels, through the development of constructive thinking and goal-achievement behaviors, as well as through planning and monitoring of adaptive strategies and, from an emotional point of view, by decreasing negative feelings towards situations and by increasing job satisfaction [24–26]. Also Griffin and colleagues find that leadership vison can promote behavioural changes, in particular work adaptivity and proactivity [21]. Anyway, in summary, we consider individual adaptive performance as the behavior exhibited by employees when they respond to and manage significant changes within their work environment. This includes adapting to new tasks, processes, technological advancements, and shifting roles. Adaptive performance is characterized by behaviors such as effectively learning new skills, creatively solving problems, handling unexpected situations, and successfully navigating interpersonal dynamics under change. These behaviors are essential in ensuring that individuals can continue to perform effectively in dynamic and evolving workplaces [16,17].

#### Leadership styles: Literature overview and the relationship with job adaptive performance

Studies on leadership span from approaches that focus on a leader’s intrinsic aspects, which support the existence of personality traits that are positively related to group performance [27], to approaches that emphasize a holistic vision of leadership, where not only the characteristics of the leader him/herself are taken into consideration, but also those of the collaborators, including the nature of their professional tasks, the goals to be achieved and the overall work situation [28]. The focus of the most recent perspectives has also been on the characteristics displayed by organizational members and on the leadership process, where leaders and followers mutually influence one another. These leader-member relationships affect an organization’s outcomes, which can include efficacy and job performance [29].

#### Neo-charismatic theories: Transactional and transformational leadership

Transactional and transformational theories, for example, study those leadership’s strategic aspects that affect performance efficacy. While the transactional style focuses on planning, supervision and evaluation of team members’ performance through a system of rewards and punishments, the transformational theory emphasizes a leader’s charisma as a personal quality of someone who is able to promote followers’ loyalty to the organization and to balance an individual’s wellbeing with that of the organization [22,23,30,31]. Since adaptivity is usually not imposed “from the top” but emerges from the bottom, transactional leaders are likely to contribute to the creation of a context that is conducive to adaptive behaviors, by clearly specifying and communicating performance expectations [32]. However, this seems to leave little executive autonomy to workers and it is the reason why there are few studies on the transactional leadership style and AP [33].

On the other hand, among contextual antecedents of AP, transformational leadership is one of the most investigated styles in literature [23,31,34]. Vera and Crossan, found that this style was particularly effective in situations of uncertainty, unpredictability and highly changing contexts, because it helped to create an organizational culture that valued adaptability and risk assumption by the members [35].

#### The emergent approaches: Servant, inclusive, authentic, humble and empowering leadership

These emerging forms of leadership focus on ethical and moral aspects, interpersonal dynamics and how this relationship could translate into positive results, in relation to conformity with organizational objectives, increase in motivational aspects and pro-social behavior [36].

Servant leadership has been one of the most studied emerging leadership types [37–39]. Greenleaf, who was the first to develop the construct [40], argued that a servant leader has the natural predisposition to put followers’ needs before personal or organizational ones. Moreover, empathy, altruism and interest in the community are the elements that lead a servant leader’s actions [41]. The desire to help collaborators should not be confused with a servile attitude; what motivates servant leaders is their decision to put others before themselves, supporting the personal and professional growth of the latter through the exercise of leader power [42]. Concerning performance, servant leaders understand that effectiveness on performance largely depends on the degree of the followers’ involvement and motivation, and that the use of transparent, ethical and persuasive communication is functional to the enrichment of relationships and to the achievement of positive long-term results with the group [38,39,43–45].

Employees’ involvement is the principal feature of the inclusive leadership style [46]. Despite the difference in status, the inclusive leader is open and available, and his/her relationships with colleagues are friendly. Additionally, this type of leader values his/her colleagues’ differences, ideas and propositions; encourages them to share knowledge and expresses divergent thoughts thus contributing to consolidating the team’s sense of belonging and a safe work environment.

Accessibility, which is one of the hallmarks of authentic leadership, is representative of other forms of positive leadership, including the transformational, the servant and the ethical [47,48]. The authentic leader is one who has the ability to gain his/her followers’ respect by way of reliability, credibility and transparency, and he/she is functional to the establishment of an organizational culture that is based on transparency [48,49].

Recently the studies have focus on the concept of humility. Similarly to previous styles, the humble leadership is a collaborators-centered approach in which leader is empathic, interested in members growth, recognizing own personal limitations and appreciate collaborators contribution [50]. It is interesting to note that scholars found that this style contribute to promote the employee’ initiative both at individual and collective level, increasing proactive behaviors [50–52].

Finally, even if the empowering leadership focus on organizational results, this style was included in the emergent approaches because the leader creates an environment where responsibilities given to collaborators increase and where individual expression is encouraged, as well as a collaborative climate, collective decision-making and sharing of knowledge within the group [53–57]. For these reasons, this style is associated with positive individual and group outcomes, with an increase in group creativity as well as with adaptability and autonomy [53].

#### Members’ leadership: shared and self-leadership

Companies show more interest in a multidisciplinary approach that promotes teamwork and this legalized the birth of an alternative model of leadership that, from a collective point of view, recognized the importance of the actions of all members.

Differently from leadership focused on a single figure, the leadership distributed among two or more individuals called shared leadership, is another perspective that meets the trend of a flat organizational structure, which is much less based on hierarchy and more centered on the transversality of roles and on skills’ overlapping. The core of shared leadership style is the interaction among group members and their mutual influence [58,59]; this social network leads them to work in a coordinated way to achieve the team’s organizational goals [60] and contribute to the improvement of complex task performance [58]. Some authors believed that group performance had improved because of shared leadership, as opposed to a single-figure leadership style [61,62] and that this positively affected the adaptive collective performance at the team level [62].

If the shared leadership is centered on collective and interactive dimensions, self-leadership is focused on processes of behavior monitoring, control and regulation, to achieve organizational goals [25] that allow a person to understand whether his/her performance falls within prefixed standards and help to keep his/her motivation high [63].

The principal features of leadership styles mentioned above are summarized in Table 1.

**Table 1:** Summary of principal leadership stiles mentioned in literature background paragraph.

Leadership style	Brief definition
**Neo- Charismatic Theories**	
**Transactional leadership**	The leader plans and supervises subordinates’ activities and make the performance evaluation using two different strategies: contingent reward or punishment and exception management [22,64,65].
**Transformational leadership**	Charismatic, inspirational, and visionary leadership aims to promote changes and trigger a trust process capable of transforming the followers’ personal goals into the organizational ones [22,64,65].
**The Emergent Approaches**	
**Empowering leadership**	Leadership as process of power sharing with followers. Leader, together with followers, outlines meanings of works, provide decision-making autonomy, and express interest in followers’ abilities [54,66]. Dimension: leading by example, coaching, Participative decision-making, informing, showing concern/interacting with the team [67].
**Authentic leadership**	Leadership is based on reliability, credibility, and transparency; the leader recognizes individual differences and values followers’ abilities using genuine communication [47–49,68,69].
**Servant leadership**	The leader puts collaborators’ needs first and “*goes beyond one’s self-interests*” [40]. Dimensions: empowerment, accountability, humility, authenticity, courage, forgiveness, stewardship, and standing back [70].
**Inclusive leadership**	The leader encourages followers’ contributions and helps them to “*overcome the inhibiting effects of status differences*, *allowing members to collaborate in process improvement*” [46] .
**Humble Leadership**	Leadership based on humility, leader self-awareness, teachability/ openness to feedback and others’ appreciation and consideration [52]
**Leader Members Exchange theory (LMX)**	Is the first leadership theory that highlight that the organizational results depend on quality of relationships between leader and members [71].
**Member’s leadership**	
**Shared leadership**	Power is distributed among members who collaborate and alternate in leadership behaviors according to the nature of the task, the organization, and their abilities [62]
**Self-leadership**	Leadership as a personal process of self-observation, self-regulation, and self-motivation "*through which the individual influences and controls his own behavior*, *knowledge and motivation in the workplace*" [25,72].

Starting from the aforesaid assumptions, this systematic review intends to: a) explore what forms of leadership included in recent literature are considered as antecedents of adaptive performance; b) understand and deepen their relationship with adaptivity in the workplace.

## Materials and methods

### Search strategy

In order to identify the relationship between leadership and adaptive performance, a comprehensive systematic literature review was conducted following Davis and colleagues recommendations for systematic review and metanalysis in social sciences [73]. To locate relevant studies, we used multiple electronic databases including Scopus, Web of Science, APA PsychINFO and Emerald Insight databases. The databased’ exploration ended in February 2024. Search keywords were “adaptive performance” OR “adaptivity” AND “leadership”, and the Boolean operators we used were OR and AND in showed search combination. The search results included articles containing the above words in the title, abstract or keywords.

To minimize the reproducibility bias and ensure that the selected articles assessed the constructs of AP and Leadership, it was decided to not use the terms of “adaptive ability”, “adaptive expertise” and “adaptability”, as synonyms of adaptive performance, because, based on previous exploratory research, it emerged that the above terms mainly referred to cognitive aspects, personality traits, skills, attitudes and individual predisposition to adaptation [74,75]. This review aimed to focus on behavioral aspects of adaptivity in the workplace and both adaptive performance and adaptivity terms are the constructs that best highlight the behavioral aspects that are used to cope with work changes [16,17,19]. Therefore, as previously indicated, we consider adaptive performance as behavior and not from an ability perspective.

### Eligibility criteria

The research team agreed into locate and select the studies that investigated the relationship between AP and leadership and that had been published in peer-reviewed scientific journals. Since the term “adaptive performance” appeared for the first time in 1999 [13], no restrictions on the year of publication were placed; furthermore, all the articles were relatively recent and none of the selected articles had a publication year prior to 2010. To select the studies to include in this review, researchers decided to follow the Preferred Reporting Items for Systematic Reviews and Meta-Analyses (PRISMA statements) guidelines [76].

The inclusion criteria were articles: (a) written in English, (b) published in peer-reviewed scientific journals or (c) conference papers, (d) that reported studies with quantitative measurements and correlation indexes of leadership style and AP, (e) featuring measurement instruments specifically designed to assess the variables of interest, and (f) that contained studies conducted in public or private organizations.

The exclusion criteria were: (a) studies not published in scientific journals, such as thesis reports or books; (b) theoretical qualitative or review articles; (c) articles reporting studies conducted in scholar contexts, measuring scholars’ adaptive performance; (d) studies assessing qualitatively AP and leadership style (e) or that quantitatively evaluated either one of it alone; (f) studies that did not measure the relationship between the two constructs and, finally, (g) the duplicate of articles found in different databases.

### Study selection

**Fig 1:**
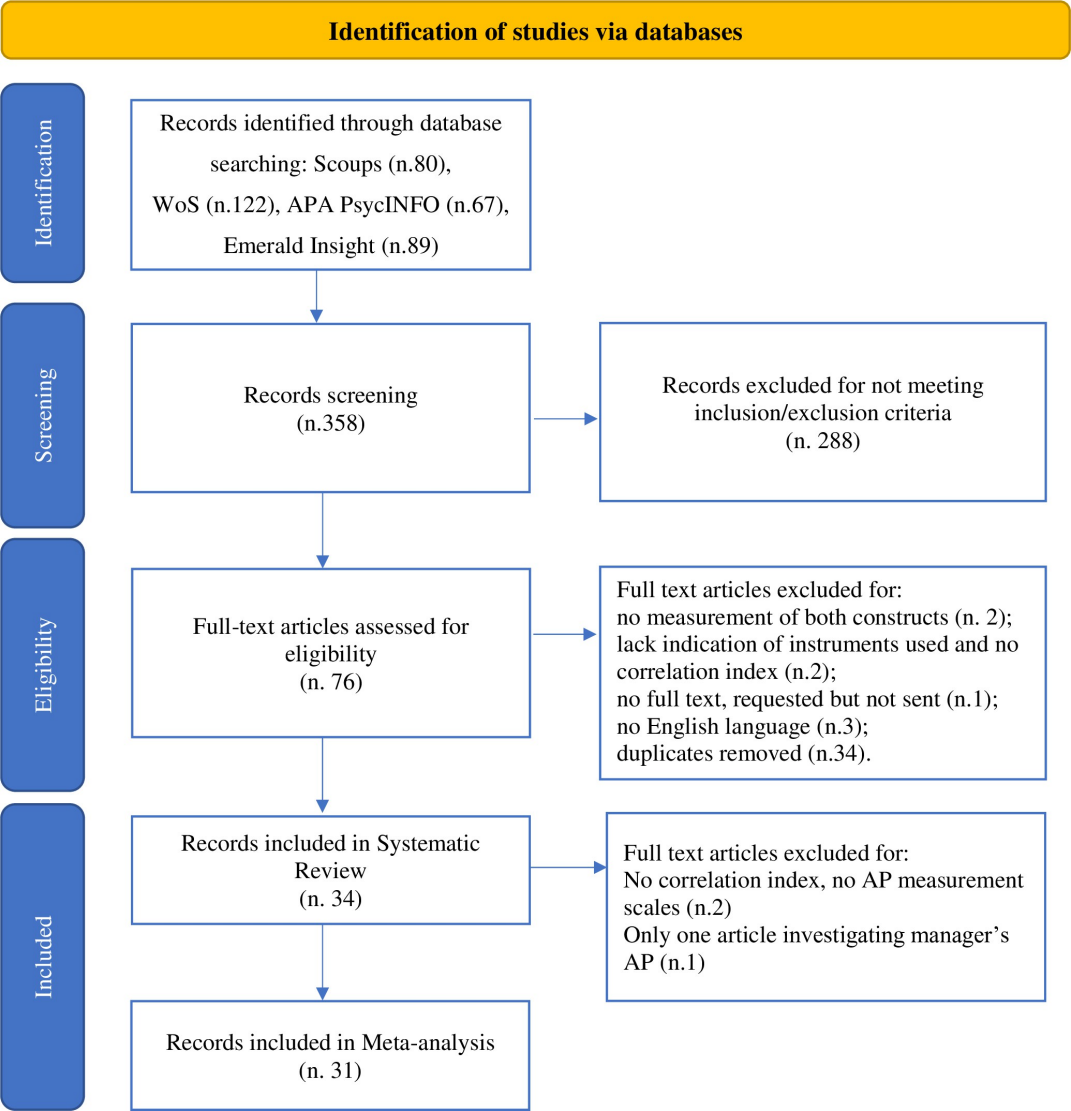
PRISMA flow chart of the search process.

The study selection was conducted by one author (AB) screening the title, abstract and keywords. A total of 358 articles were found through this literature research. The application of the eligibility criteria previously described reduced the number of articles to 76. Then, the first and the second author (CP), checked and reviewed the studies included on this first step. They agreed that 34 papers were deemed suitable for the review and 31 for the meta-analysis. The third author (MGM) supervised the process. Only two articles elicited indecision with respect to include or exclude it form the review. So, we calculated the Kappa score to estimate the level of agreement intra judges and it indicated an almost substantial agreement between the two authors (k = 0.725–95,24% of agreement). Fig 1 PRISMA flow chart representing the described screening process.

### Data collection and coding

Two reviews revised any paper retrieved independently to check for agreement and increase the validity of the study coding. Then, they used Microsoft Excel 2019 program (For Mac version) to organize data extraction categorizing and together they decided to divided and classified each article selected for the review according to the leadership style investigated (see Table 2). Any discrepancies in reviewers’ classification were resolved by discussion.

**Table 2:** Studies included in the systematic review, grouped according to the different leadership style investigated.

Leadership style (nr. of items using it)	References
**Neo- Charismatic Theories**	
**Transactional leadership (2)**	Hoandră (2017) [33] AlAbri et al. (2022) [77]
**Transformational leadership (7)**	Curado & Santos (2021) [78]
	Adams & Webster (2021) [10]
	Charbonnier-Voirin et al. (2010) [23]
	Pratoom (2022) [79]
	Wang et al. (2017) [80] Tung & Shih (2023) [81] Fan (2023) [82]
**The Emergent Approaches**	
**Coaching style (1)**	Hui & Sue-Chan (2018) [83]
**Voice with supervisors (1)**	Huntsman et al. (2021) [84]
**Empowering leadership (5)**	Rousseau and Aubé (2020) [85]
	Yang et al. (2017) [57] Sanchez-Manzanares et al. (2020) [86] Huntsman et al. (2022) [87] Xu & Zhang (2022) [56]
**Employee oriented leadership (1)**	Lichtenthaler and Fischbach (2018) [88]
**LMX (1)**	Xu & Zhang (2022) [56]
**Authentic leadership (1)**	Kaya & Karatepe (2020) [89]
**Servant leadership(5)**	Kaltiainen & Hakanen (2022) [90]
	Kaya & Karatepe (2020) [89]
	Bande et al. (2016) [8] Zia et al. (2023) [91] Balti & Karoui Zouaoui (2023) [92]
**Inclusive leadership (3)**	Qurrahtulain et al. (2020) [93]
	Bataineh et al. (2022) [94]
	Yu (2020) [95]
**Humble Leadership (2)**	Roshayati (2023) [96] Zhang et al. (2024) [97]
**Member’s leadership**	
**Shared leadership (4)**	Rousseau and Aubé (2020) [85]
	Fu et al. (2020) [98] Han et al. (2021) [99] Tung & Shih (2023) [81]
**Self-leadership (4)**	Marques-Quinteiro et al. (2019) [63]
	Marques-Quinteiro & Curral (2012) [100]
	Hauschildt & Konradt (2012) [24] Maden-Eyiusta & Alparslan (2022) [101]
**Others Theories**	
**Directive leadership (1)**	Sanchez-Manzanares et al. (2020) [86]
**Paradoxical leadership (2)**	Sparr et al. (2022) [102] Li & Ding (2022) [103]
**Leader Adaptive Personality (1)**	Bajaba et al. (2021) [104]
**Laissez-faire Leadership (1)**	Tung & Shih (2023) [81]

In total 34 articles were selected; some references were reported several times because these studies had investigated more types of leadership style (Rousseau and Aubé, 2020 [85]; Kaya and Karatepe, 2020 [89]; Sanchez-Manzanares et al., 2020 [86]; Tung & Shih, 2023 [81]; Xu & Zhang, 2022 [56]).

Due to the heterogeneity of the studies found in the articles that had been selected, we chose to combine a qualitative narrative approach and a quantitative meta-analysis to explain the leadership and AP relationship.

Principal descriptive information of the studies included in review are reported in the follow paragraph titled: “Results: Article Description” and synthetized in Table 3 where Pearson’s r correlation coefficients were reported and used to determine the effect size for the meta-analysis (see Table 3).

**Table 3:** Main characteristics of the studies included in meta-analysis.

	Ref.	Leadership Style Dimensions	AP Dimensions	N	Mean age (years)—SD	% of women	Leadership scale	Leadership Evaluation	AP Scale	AP Evaluation	Effect size [r or ß correlation coefficient]
1	Kaltiainen and Hakanen (2022) [90]	Servant (T1)	Adaptive Stress Management (T2)	2453	48.4(9.6)	85.5%	van Dierendonck and Nuijten (2011) [70]	Auto- evaluation	Charbonnier-Voirin and Roussel (2012) [20]+ 1 item ad hoc	Auto- evaluation	**.19 p < .001[r]**
2	Kaltiainen and Hakanen (2022) [90]	Servant (T1)	Adaptive Reactivity (T2)	2453	48.4(9.6)	85.5%	van Dierendonck and Nuijten (2011) [70]	Auto- evaluation	Charbonnier-Voirin and Roussel (2012) [20] + 1 item ad hoc	Auto- evaluation	**.12 p < .001[r]**
3	Kaltiainen and Hakanen (2022) [90]	Servant (T1)	Adaptive Creativity (T2)	2453	48.4(9.6)	85.5%	van Dierendonck and Nuijten (2011) [70]	Auto- evaluation	Charbonnier-Voirin and Roussel (2012) [20] + 1 item ad hoc	Auto- evaluation	**.07** **p < .01[r]**
4	Kaltiainen and Hakanen (2022)[90]	Servant (T1)	Interpersonal Adaptivity (T2)	2453	48.4(9.6)	85.5%	van Dierendonck and Nuijten (2011) [70]	Auto- evaluation	Charbonnier-Voirin and Roussel (2012) [20] + 1 item ad hoc	Auto- evaluation	**.16 p < .001[r]**
5	Curado and Santos (2021) [78]	Transformational	Dealing With Emergency and Crisis	192	range	63.02%	Bass e Avolio (1995) [105]	Hetero-evaluation	Charbonnier-Voirin and Roussel (2012) [20]	Auto- evaluation	** .021** **p < .05 [ß]**
6	Curado and Santos (2021) [78]	Transformational	Training And Learning Efforts	192	range	63.02%	Bass e Avolio (1995) [105]	Hetero-evaluation	Charbonnier-Voirin and Roussel (2012) [20]	Auto- evaluation	** .016** **p < .05 [ß]**
7	Curado and Santos (2021) [78]	Transformational	Interpersonal Adaptability	192	range	63.02%	Bass e Avolio (1995) [105]	Hetero-evaluation	Charbonnier-Voirin and Roussel (2012) [20]	Auto- evaluation	** .108** **p < .05 [ß]**
8	Rousseau, V., & Aubé, C. (2020) [85]	Empowering	Team Adaptive Performance	82	38.55(7.95)	23.62%	Arnold et al. (2000) [67]	Hetero-evaluation	Griffin & Hesketh (2003) [106]	Hetero-evaluation	**.26** **p < .05[r]**
9	C Rousseau, V., & Aubé, C. (2020) [85]	Shared	Team Adaptive Performance	82	38.55(7.95)	23.62%	Hiller et al. (2006) [107]	Hetero-evaluation	Griffin & Hesketh (2003) [106]	Hetero-evaluation	**.32** **p < .01[r]**
10	Kaya and Karatepe (2020) [89]	Servant	Adaptive Performance	226	range	45.1%	Liden et al., 2014 [108]	Hetero-evaluation	Hartline & Ferrell (1996) [109]	Hetero-evaluation	**.731 p < .01[r]**
11	Kaya and Karatepe (2020) [89]	Authentic	Adaptive Performance	226	range	45.1%	Wong & Cummings (2009) [110]	Hetero-evaluation	Hartline & Ferrell (1996) [109]	Hetero-evaluation	**.703 p < .01[r]**
12	Qurrahtulain[93] *et al*. (2020)	Inclusive	Adaptive Performance	288	range	27%	Carmeli et al. (2010) [111]	Hetero-evaluation	Adapted from Pulakos et al. (2000) [16]	NS	**.343 p < .001[r]**
13	Marques-Quinteiro *et al*. (2019) [100]	Self-Leadership	Adaptive Performance	52	44.54(7.23)	42%	Hougthton & Neck (2002) [26] (Portoguese validation Marques-Quinteiro et al., 2012) [112]	Auto- evaluation	Marques-Quinteiro et al. (2015) [113]	Auto- evaluation	**.54 p < .001[r]**
14	Fu *et al*. (2020) [98]	Shared Leadership—Perceived Team Support	Adaptivity	301	range	45.5%	Hoch and Kozlowski (2014) [114]	Auto- evaluation	Griffn, Neal & Parker (2007) [19]	Auto- evaluation	**.576 p < .01[r]**
15	Fu *et al*. (2020) [98]	Shared leadership -Team Learning	Adaptivity	301	range	45.5%	Hoch and Kozlowski (2014) [114]	Auto- evaluation	Griffn, Neal & Parker (2007)[19]	Auto- evaluation	**.457 p < .001[r]**
16	Fu *et al*. (2020) [98]	Shared Leadership—Team-Member Exchange	Adaptivity	301	range	45.5%	Hoch and Kozlowski (2014) [114]	Auto- evaluation	Griffn, Neal & Parker (2007)[19]	Auto- evaluation	**.624 p < .01[r]**
17	Sparr et al. (2022) [102]	Paradoxical Leadership	Adaptivity	154	NS	25%	Ad hoc based on Smith & Lewis (2011) [115]	Hetero-evaluation	Griffn, Neal & Parker (2007)[19]	Hetero-evaluation	**.18** **p < .05[r]**
18	Adams and Webster (2021) [10]	Transformational Leadership	Adaptive Performance	314	35.9(0.49)	66%	Carless et al. (2000) [116]	Hetero-evaluation	Ad hoc	Auto- evaluation	**.48** **p < .01[r]**
19	Lichtenthaler and Fischbach (2018) [88]	Employee Oriented Leadership	Job Performance (Task, Adaptive and Proactive)	117	45.26(8.45)	19.66%	Wilde et al., 2009 (German version Ekvall & Arvonen, 1994)[117]	Hetero-evaluation	Griffn, Neal & Parker (2007) [19]	Hetero-evaluation	**.26** **p < .05 [r]**
20	Charbonnier-Voirin *et al*. (2010) [23]	Transformational Leadership	Adaptive Performance	120	38.5 (NS)	16%	Podsakoff, MacKenzie, Moorman & Fetter (1990) [118]	Hetero-evaluation	Charbonnier-Voirin and Roussel (2012) [20]	Auto- evaluation	**.44** **p < .01[r]**
21	Hoandră (2017) [33]	Transactional Leadership	Team Adaptive Performance	148	39.6(10.34)	49.32%	Northouse, P.G. (2001) [119]	Auto- evaluation	Marques-Quinteiro et al., 2015	Auto- evaluation	**.50** **p < .01[r]**
22	Pratoom (2022) [79]	Transformational Leadership	Adaptive Performance	480	range	88.20%	Avolio et al. (1999) [120]	Hetero-evaluation	Charbonnier-Voirin and Roussel (2012) [20]	Auto- evaluation	**.23** **p < .05 [r]**
23	Huntsman et al. (2021) [84]	Emp_Voice With Immediate Supervisors	Team Adaptive Performance	757	NS	NS	Van de Post et al. (1997) [121]	Auto- evaluation	De Waard et al. (2013) [122]	Auto- evaluation	**.20 p < .001[r]**
24	Huntsman et al. (2021) [84]	Emp._Voice With Seniors Leaders	Team Adaptive Performance	757	NS	NS	Van de Post et al (1997) [121]	Auto- evaluation	De Waard et al. (2013) [122]	Auto- evaluation	**.42 p < .001[r]**
25	Huntsman et al. (2021) [84]	Emp_Authonomy	Team Adaptive Performance	757	NS	NS	Van de Post et al (1997)[121]	Auto- evaluation	De Waard et al. (2013) [122]	Auto- evaluation	**.55 p < .001[r]**
26	Yu (2020) [95]	Inclusive	Team Adaptive Performance	171	range	45.35%	Carmeli, Reiter-Palmon & Ziv (2010) [111]	Hetero-evaluation	Han & Williams (2008 GOM) [123]	Hetero-evaluation	**.19** **p < .01[r]**
27	Marques-Quinteiro and Curral (2012) [63]	Self-Leadership Behaviour Focus On Strategies	Adaptive Performance	108	38(9.8)	47%	Hougthton & Neck, 2002 [112](Portuguese validation Marques-Quinteiro et al., 2012) [112]	Auto- evaluation	Griffn, Neal & Parker (2007)[19]	Auto- evaluation	**.13** **p < .05 [r]**
28	Marques-Quinteiro and Curral (2012) [63]	Self-Leadership Natural Reward Strategies	Adaptive Performance	108	38(9.8)	47%	Hougthton & Neck, 2002 [112](Portuguese validation Marques-Quinteiro et al., 2012)[112]	Auto- evaluation	Griffn, Neal & Parker (2007) [19]	Auto- evaluation	**.33** **p < .01[r]**
29	Hui and Sue-Chan [83](2018)	Emp._Guidance Coaching	Adaptive Performance	373	range	48.73%	Hui et al. (2013) [124]	Hetero-evaluation	LePine (2003) [125]	Hetero-evaluation	**-.10** **p < .05 [r]**
30	Hui and Sue-Chan (2018) [83]	Emp._Facilitation Coaching	Adaptive Performance	373	range	48.73%	Hui et al. (2013) [124]	Hetero-evaluation	LePine (2003) [125]	Hetero-evaluation	**.14** **p < .05 [r]**
31	Hauschildt and Konradt (2012) [24]	Self-Leadership	Individual Adaptability	81	33.4(8.67)	60.49%	Hougthton & Neck (2002)[26]	Auto- evaluation	Griffn, Neal & Parker (2007) [19]	Auto- evaluation	**.37 p < .001[r]**
32	Hauschildt and Konradt (2012) [24]	Self-Leadership	Team Adaptability	81	33.4(8.67)	60.49%	Hougthton & Neck (2002) [26]	Auto- evaluation	Griffn, Neal & Parker (2007)[19]	Auto- evaluation	**.44 p < .001[r]**
33	Bande *et al*. (2016) [8]	Servant	Adaptability	290	42.20(8.5)	19.66%	Ehrhart (2014)[126]	Hetero-evaluation	Griffn, Neal & Parker (2007)[19]	Hetero-evaluation	**.23** **p < .05 [ß] **
34	Wang *et al*. (2017) [80]	Transformational	Adaptability	185	40.57(12.22)	32.70%	Podsakoff et al. (1990) [118]	Hetero-evaluation	Van der Heijde & Van der Hijden, (2006) [127]	Hetero-evaluation	**.24** **p < .01[r]**
35	Bataineh *et al*. (2022) [94]	Inclusive	Adaptive Performance	169	range	66,9%	Carmeli, Reiter-Palmon & Ziv (2010) [111]	Hetero-evaluation	Charbonnier-Voirin and Roussel (2012) [20]	Auto- evaluation	**.26** **p< .05 [ß] **
36	Yang *et al*. (2017) [57]	Empowering	Adaptive Performance	420	range	57,9%	Wang et al., (2008) [128]	Auto- evaluation	Tao & Wang (2006) [129]	Auto- evaluation	**.59** **p < .01[r]**
37	AlAbri et al. (2022) [77]	Transactional Leadership	Adaptive Performance	233	range	57,10%	NS	NS	Pradhan & Jena (2017) [130]	Auto- evaluation	**.473** **p< .01 [r]**
38	Li & Ding (2022) [103]	Paradoxical Leadership	Adaptive Performance	519	range	52,41%	Zhang et al. (2015) [131]	Hetero-evaluation	Griffn, Neal & Parker (2007) [19]	Auto- evaluation	**.449** **p < .001[r]**
39	Huntsman et al. (2022) [87]	Empowering Leadership (immediate supervisor)	Adaptive Performance	1113	NS	NS	Fire Industry Organizational Culture Survey (FIOCS) Huntsman and Greer (2019b) [132]	Hetero-evaluation	De Waard et al. (2013)[122]	Auto- evaluation	**.29** **p < .001[r]**
40	Huntsman et al. (2022) [87]	Empowering Leadership (Senior supervisor)	Adaptive Performance	1113	NS	NS	Fire Industry Organizational Culture Survey (FIOCS) Huntsman and Greer (2019b)[132]	Hetero-evaluation	De Waard et al. (2013) [122]	Auto- evaluation	**.51** **p < .001[r]**
41	Xu & Zhang (2022) [56]	Empowering Leadership	Adaptive Performance	292	33.11	65%	Ahearne et al. (2005) [133]	Hetero-evaluation	Zhang & Quanquan (2009) [134]	Auto- evaluation	**.14** **p < .01[r]**
42	Xu & Zhang (2022)[56]	LMX	Adaptive Performance	292	33.11	65%	LMX-7 Graen & Uhl-Bien (1995) [135]	Hetero-evaluation	Zhang & Quanquan (2009)[134]	Auto- evaluation	**.20** **p < .001[r]**
43	Zia et al. (2023)[91]	Servant Leadership	Adaptive Performance	318	range	36%	Liden et al., 2014 (short version)[108]	Hetero-evaluation	Kaya & Keratepe (2020)[89]	Hetero-evaluation	**.52** **p < .01[r]**
44	Fan (2023) [82]	Transformational Leadership	Adaptive Performance	300	NS	NS	Li-Chaoping (2005) [136]	Hetero-evaluation	Qi & Zhongming (2006) [137]	Auto- evaluation	**0.475** **p < .01[r]**
45	Tung & Shih (2023)[81]	Transformational Leadership	Adaptive Performance	68	NS	19,20%	Carless et al. (2000)[116]	Hetero-evaluation	Han & Williams (2008 GOM) [123]	Hetero-evaluation	**.26** **p < .05[r]**
46	Tung & Shih (2023) [81]	Shared Leadership (Decentralization)	Adaptive Performance	68	NS	19,20%	Chiu et al. (2016) [138]	Hetero-evaluation	Han & Williams (2008 GOM)[123]	Hetero-evaluation	**.14** **Not sing.**
47	Tung & Shih (2023) [81]	Shared Leadership (Density)	Adaptive Performance	68	NS	19,20%	Chiu et al. (2016) [138]	Hetero-evaluation	Han & Williams (2008 GOM)[123]	Hetero-evaluation	**.06** **Not sign.**
48	Roshayati (2023) [96]	Humble Leadership	Adaptive Performance	200	NS	58,50%	Owens et al. (2013) [139]	Hetero-evaluation	Koopmans et al. (2013) [140]	Auto- evaluation	**.366** **p < .000 [ß] **
49	Zhang et al. (2024) [97]	Humble Leadership	Adaptive Performance	201	range	56,50%	Owens et al. (2013) [139]	Hetero-evaluation	Griffn, Neal & Parker (2007) [19]	Hetero-evaluation	**.61** **p < .001[r]**
50	Maden-Eyiusta & Alparslan (2022) [101]	Self-leadership	Task adaptivity	174	39.95 (6.99)	62%	(ASLQ; Houghton et al., 2012)[141]	Auto- evaluation	Griffn, Neal & Parker (2007)[19]	Auto- evaluation	**.33** **p < .01[r]**
51	Maden-Eyiusta & Alparslan (2022) [101]	Self-leadership	Task adaptivity	135	36.27 (8.82)	51%	(ASLQ; Houghton et al., 2012)[141]	Auto- evaluation	Griffn, Neal & Parker (2007)[19]	Hetero-evaluation	**.57** **p < .01[r]**
52	Maden-Eyiusta & Alparslan (2022)[101]	Self-leadership	Task adaptivity	135	36.28 (8.83)	51%	(ASLQ; Houghton et al., 2012)[141]	Auto- evaluation	Griffn, Neal & Parker (2007)[19]	Hetero-evaluation	**.48** **p < .01[r]**

For those studies did not shows a global score of AP or leadership style, the outcomes were reported separately for each dimension. NS = Not Specified.

## Results: Articles description

All articles contained studies that investigated the relationship between leadership and adaptive AP; some of them read AP at the individual level (n. 24), while others measured it at the team level (n. 6) or at both levels (n. 3). Han and Williams, who studied the differences between individual performance and team adaptive performance, found that the two constructs were closely related, concluding that a high level of individual adaptivity extended to the team through members’ coordination and cooperation capacity.

Except for the studies of Kaltiainen and Hakanen [90] and Curado and Santos [78], which detected AP as a multidimensional construct, all studies explored AP as a mono-dimensional construct and the scales mostly used to assess job adaptivity were: Griffin, Neal and Parker’s scale [19] and Charbonnier-Voirin and Roussel’s scale [20].

As for the sample, the majority of the articles reported surveys that had been carried out on workers; only two surveys had collected data from students [80,86]. All of the studies were carried out in one or more organizations, with the exception of the one by Sanchez-Manzanares and colleagues [86], who conducted their research in an artificial context of simulation. In most of the cases, nr. 13, data were collected from private companies, 8 studies are conducted in public sector and 2 studies in enterprises of both sectors, private and public. With respect to the type of organizations involved, it ranges from textile and manufactory industry, bank financial and accounting firms, ICT/electronic firms, health care and human service sector organizations and hospitality industries. One article used an online crowdsourcing platform (MTurk) to collect data and 10 articles did not present sufficient elements for us to understand what type of organizations the data were collected from.

Finally, all the selected articles were recent, with the year of publication ranging from 2010 to 2024, and most of the studies was conducted in Europe (n. 12), followed by Asia (n. 17), North America (n. 4) and Africa (n.1).

### Leadership and adaptive performance: The relationship

Many of the studies included not only additional designs aimed to analyze the primary relationship of interest, but also the covariate and moderators’ effects. Of the 34 articles included in our systematic review, the majority (n. 33) probed the relationship between a specific leadership style and employee AP and one focused on leader’s personality and leader’s adaptivity.

All articles we selected showed studies with a statistically significant relationship between leadership and AP (see Table 3). Regarding the direction of that relationship, only one study revealed a negative correlation between guidance coaching style and AP. As hypothesized by the authors, this could be because people who had received guidance coaching were “*less able to adjust their behaviors to respond to changed tasks and/or job environment”*, as opposed to facilitator coaching, which encouraged the exploration and active learning that would help to cope with new experiences [83].

Seven articles included in our review linked transformational leadership with AP. As previously mentioned, this adaptation-facilitating style is one of the most widely studied in literature.

Two articles studied the subject of transformational leadership and AP in healthcare companies [78,82] by using respectively job satisfaction and organizational identification as principal mediators. Both founded the same positive results of transformational leadership on healthcare operators’ AP. Adams and Webster (2021), on the other hand, explored transformational leadership during the Covid-19 pandemic. They included the aforesaid leadership style as a control variable and took into consideration the leader’s gender, as a moderator in the relationship between task or relationship-oriented leadership and adaptivity [142]. The authors connected transformational task-oriented leadership behaviors and a leader’s Interpersonal Emotion Management (IEM) [143] with the AP of collaborators and their confidence towards the leader. The authors found that IEM mitigated, both directly and indirectly, the impact of negative emotions that employees faced during exceptional and unexpected job demands, which is typical of AP, when they trusted their leader, especially if the leader was a woman. Conversely, task-oriented behaviors seemed to directly influence AP in crisis situations, reducing uncertainty when collaborators were given clear and precise instructions on what to do, without affecting trust between leaders and followers.

Like the previous study, Lichtenthaler and Fischbach showed that employee-oriented leadership, through job crafting, had a greater positive effect on adaptivity than on proactive and task performance. On the other hand, the actions aimed at job crafting prevention had a negative impact on both the employees’ health and their performance. Wang, Demerouti and Le Blanch (2017) found that a positive relationship between transformational leadership and AP favor job crafting, however, organizational identification seemed to decrease the strength of the leadership-AP relationship, probably because the identification with transformational leader and the identification with the organization were mutually exclusive. Charbonnier-Voirin et colleagues [23] and Pratoom [79] studied transformational leadership at the collective level, as well as transformational leadership climate, as an organizational antecedent that influence adaptivity.

Two articles detected the relationship between transactional leadership and AP. Hoandră investigate AP at the team level and results highlighted that the contingent reward component of transactional leadership could improve the discussion among group members, when a specific task was to be solved rapidly thus encouraging an adaptive approach to sharing innovative strategies for problem solving and task execution [33]. While AlAbri and colleagues proposed transactional leadership as moderator between some HR Management practices, including performance appraisal, job enlargement, employee’ involvement, job enrichment and training finding that this style moderate only the relationship between job enrichment and AP probably because it focus on punishment and rewards [77]. Also Sanchez-Manzanares and colleagues [86] showed that a directive leadership style, which is similar to the transactional one, could have a better positive influence on AP than empowering leadership style, in an emergency situation but they explained the results by attributing them to the context of the experimental simulation, the participants’ unfamiliarity with the tasks and the time constraints; all contextual factors that could improve the influence of directive leadership on AP.

The Huntsman and colleagues’ studies [84,87], on the other hand, investigated the AP of firefighters who exercised in real organizational structures where job activities usually took place in situations of urgency and emergency. The authors explored the follower empowerment practices that allowed for professional growth, autonomy and possibility of individual expression with supervisors, discovering that these elements contributed to AP even in contexts where rigid hierarchical structures persisted. Similarly, Rousseau and Aubé [85], who worked with public companies that provided public safety services, showed that empowering leadership behaviors, implemented by superiors and perceived by team members, influenced the group’s adaptivity through the development of shared leadership and Xu and Zhang [56] stated that empowering leadership influence University Teachers ‘AP individually trough the mediating role of leader-member exchange (LMX) relationship [56].

Tung and Shih paper compare transformational and lasses-faire leadership style as moderators in shared leadership and AP relationship providing evidence that shared leadership is complementary to the “top down” styles and an perception of high transformational leadership is a facilitator of team adaptivity thanks to shared leadership while laissez-faire style decrease the shared leadership and the team AP too [81].

Five papers deepen the relationship between servant leadership and adaptive performance, in particular Balti and colleagues in a recent study detect that building a “servant leadership climate” in workplace, could influence emotional intelligence and contribute to individual adaptive performance development [92]. Kaltiainen and Hakanen developed a longitudinal design demonstrating that servant leadership involved improvements in stress management, as well as responsiveness, creativity and interpersonal adaptation, thanks to work engagement. In addition, seems that servant leadership influenced salespeople’s adaptivity, whether directly or indirectly, via the increase of self-efficacy and intrinsic motivation [8]. Similarly, Kaya and Keratepe [89] found that, on hotel personnel, servant leadership had a positive direct and indirect effect, through work engagement, on AP and Fu and colleagues [98] studying the same sector, found that a shared leadership influenced adaptivity trough proactive behavior.

Also the humble leadership had a positive relationship with AP on employee that work from remote [96] and the relationship among two constructs was mediated by self-determination [97].

Regarding self-leadership, Hauschildt and Konradt’s study [24] show a positive relationship both on team and individual AP and by Maden-Eyiusta and Alparslan [101] in their cross sectional and longitudinal studies in which they find a positive indirect effect of self-leadership on work from home employee task adaptivity trough psychological empowerment. Additionally, Marques-Quinteiro and colleagues [100], conducting a quasi-experimental research design in a bank during a crisis period, observed a positive relationship between self-leadership and individual AP, thanks to self-regulation strategies that had contributed to performance improvement. In particular, the results of a study that Marques-Quinteiro and Curral [63] carried out in a technology company, revealed that behavior-centered self-leadership strategies did not necessary promote AP. The explanation provided was that goal-focused strategies could be more functional to qualified and specialized human resources working in technological sectors, where high standards of performance are required, along with innovativeness and ability to anticipate changes. The coaching actions implemented by supervisors could increase the auto-regulation mechanisms, discovering that “facilitation-based coaching” had a positive effect on AP because it encouraged active exploration and self-learning strategies that could be used in new situations [83].

On the assumption that one of the elements that characterizes the organizational context is the increase in ambivalent job demands, Sparr, Knippenberg and Kearney [102] defined the construct of “*paradoxical leadership*” as the leader’s ability to balance directive and participative approaches and to make sense of opposite job demands. The authors demonstrated that paradoxical leadership helped people be predisposed to change, adaptivity and proactivity, through the mediation of *change readiness*.

Finally, only the study of Bajaba and colleagues [104] investigated the manager’s AP during the Covid-19 pandemic, arguing that leaders that had an adaptive personality adopted an adaptive attitude and were able to anticipate and make the necessary changes to help teams and collaborators deal with emergencies.

### Qualitative discussion

All previously mentioned studies investigated in depth the strength of the relationship between leadership and AP, and our systematic review confirmed the positive influence that different styles of leadership have on adaptivity and proactive behaviors towards change.

A first consideration is that the influence of a leadership style could be related to the work sector. The servant style predominated in studies conducted in hotel management and in sales [8,90], where the achievement of organizational goals passed through the relationship with customers.

On the contrary, in emergency work situations requiring team rapidity and coordination, a directive approach not only allows employees to maintain high standards of performance, but also supports adaptivity [84,86]. Additionally, task-focused behaviors and contingent reward mechanisms are effective during crisis and in situations where there is a need for creative, yet quick, problem solving [10].

Our second consideration has to do with the role of individual and organizational mediators. At the individual level, the Self-leadership style and Self-regulation mechanisms, in fact, helped reduce negative perceptions and related resistances; furthermore, it directed attention on positive aspects that allowed for the development of constructive mental patterns, where planning and monitoring personal behaviors helped people adapt to changes [144].

Some individual factors, like work engagement, job satisfaction, intrinsic motivation, vigor at work, absorptive capacity and self-efficacy, emotional intelligence, suggested that adaptivity was promoted by leadership as a motivational key. The transformational leader, for example, stimulates and encourages the use of new skills by creating an attractive vision of the future, inspiring their followers to take responsibilities and engage in extra role behaviors.

Another aspect was related to the shared assumption of leadership which seems to create the right conditions to facilitate adaptivity to new job demands. Relationship-oriented shared leadership, for example, had a direct and positive effect on job performance [99]. The opportunity for members to share leadership behaviors created a supportive climate that promoted proactivity and, in turn, stimulated adaptivity. Furthermore, by encouraging mutual trust, members developed a psychological safety net that was ideal for individual initiative and it encouraged group discussion on goals, strategies, processes and how to cope with new, unpredictable or paradoxical situations [85,98].

## Meta-analysis: Effect coding and meta-analytical procedure

In order to statistically measure the relationship between leadership and AP, the effect sizes from 31 of the 34 articles that comprised our systematic review were included in the meta-analysis, supplying 52 different effect sizes from 32 samples (see Table 3).

Samples ranged from 52 to 2,453 participants, including 11,640 people in total. The mean percentage of women across the studies was 47% (k = 29) and the mean age across studies was 39.65 years (*SD* = 4.52, only 13 papers reported this information). The Pearson correlation coefficient was considered as effect size. When global score of meta-analyzed constructs were not reported, we considered correlations among subscales as effect size.

We also planned to test moderation effect on the relationship between leadership and AP. More precisely, we considered 9 potential moderators, based on the assessment of three independent judges (two authors and one researcher) who reviewed the articles.

Firstly, we considered a “leadership group” as a moderator; we divided leadership into three macro groups, according to the literature [145], so to test the effect on AP: neo-charismatic theories, emergent approaches and members’ leadership.

The other 3 moderators were related to the measurement of AP and leadership: levels of AP measured (at the individual level vs the team level); the evaluation of AP (auto vs hetero) to differentiate between job performance measured by leader and by collaborators themselves; and leadership evaluation (auto-evaluated by managers or hetero-assessed by their followers).

We also included organizational features as moderators. We considered whether the study was conducted in a private or public organization, and the job sectors of the companies involved in the studies, divided into 4 macro-sectors: healthcare and human services; market services; manufacturing industries; and, finally, the mixed sector, which encompassed studies involving more than one organization belonging to different job sectors.

We also included organizational changes as moderators, since AP was tied to one’s ability to adapt to work variations [16,17]. Finally, we considered the research design (cross sectional vs longitudinal) and the coefficients (ß vs r).

To conduct our meta-analysis, we considered Pearson’s r as effect size. In three studies (5 effect sizes), the Pearson correlation coefficient was not reported, and regression coefficients were used and transformed into r following the Peterson and Brown’s (2005) formula. Correlation coefficients were corrected for small sample bias and then transformed into Fisher’s *Z*_*r*_. We performed a 3-level random meta-analysis that would take into account dependency among effect size [146,147]. More precisely, level 1 and level 2 referred to people nested in effect sizes and represented levels of classical meta-analysis. We added a further nesting level, considering a sample in which effect sizes were nested. This approach enabled us to take into account dependency across effect sizes coming from the same sample.

For moderation analysis purposes, we used a meta-regression procedure using dummy coding in case of categorial moderator with more than two levels. All meta-analytical procedures were done in R (R Core Team, 2021) and with the metafor Package [148].

### Publication bias

Given the structure of analysis, we used a generalization of Egger’s regression test, so to test for publication bias. More precisely, we meta-regressed our outcome on two measures of precision, namely standard error of effect size and reciprocal of sample size.

## Meta analytical results

### Pooled effect

The overall effect was significant, *Z*r = .39, SE = .04, p < .001. 95%CI [.32, .47], r = .37, indicating that leadership and AP were significantly and moderately correlated to each other. Influence analysis revealed that the effect size changed little (range Zr = 0.38 to 0.40) after every single study was excluded from the analysis.

**Fig 2:**
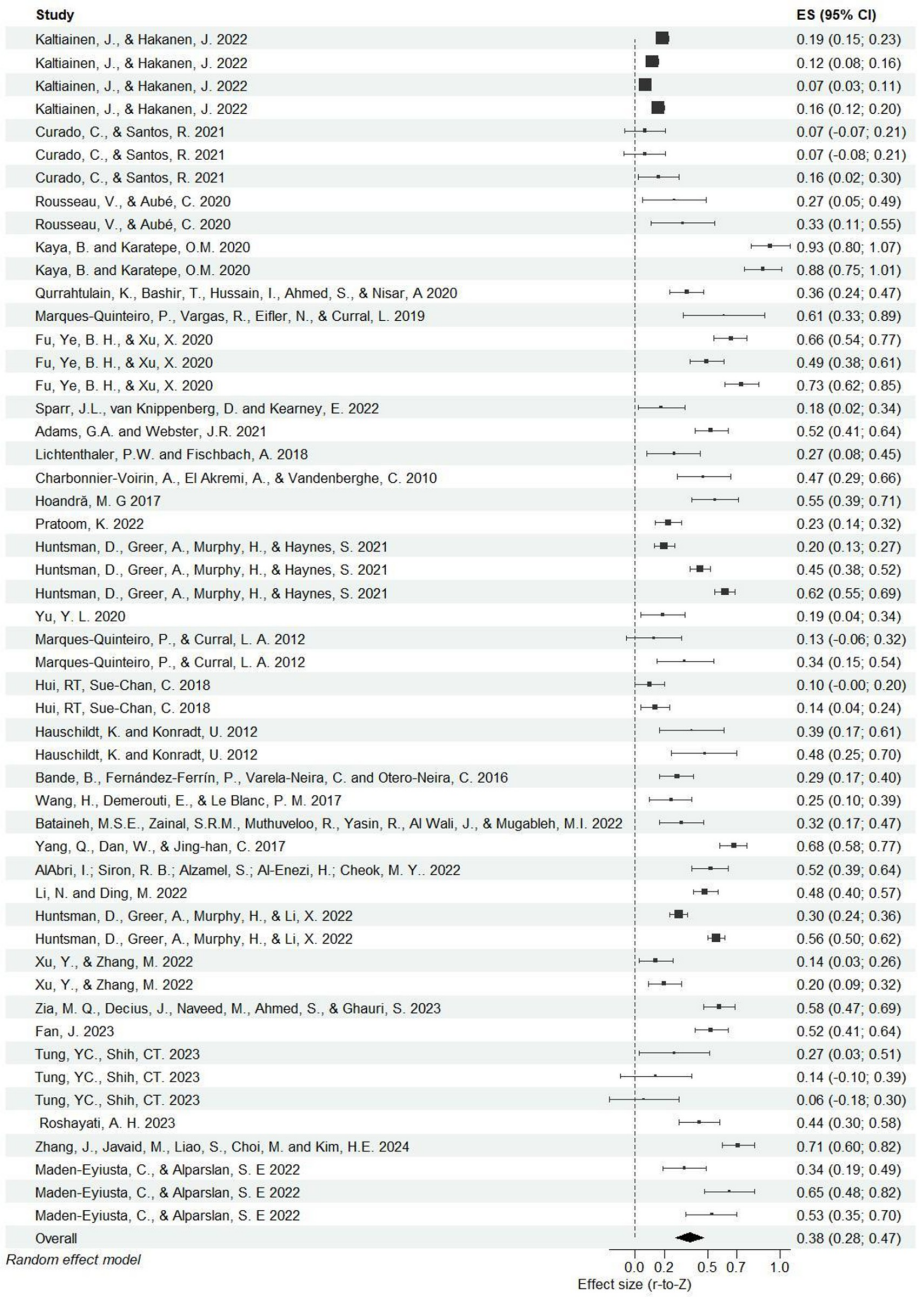
Forest plot displaying individual and pooled effect sizes (95% confidence intervals) of the studies relating to AP and leadership style included in the meta-analysis. Box sizes represent the weight of each study in meta-analysis.

However, heterogeneity in effect sizes was high, Q(df = 51) = 1045.34, p < .001, I^2^ = 94.78%, which was mostly due to variance between samples (71.22%) and between effect size (2.3.56%). This indicates that the strength of the relationships between leadership and AP was variable across studies and suggesting that moderation would have occurred (see forest plot in Fig 2).

### Moderation analysis

Table 4 reports our moderation analysis along with significance of effect of each moderator. As indicated, the only significant moderation was due to the kind of effect size considered, for which Pearson’s r tended to supply higher values than beta. It is worth noting that, self-evaluated leadership was more correlated with AP than hetero-evaluated leadership was, albeit this difference did not reach statistical significance. No other significant moderation appeared.

**Table 4:** Categorical moderation analysis.

Moderators	Zr (95%CI)	SE	k	Moderation effect
**Leadership group**				F(2, 47) = 0.01, p = .99
Neo Charismatic Theories	0.40 (0.25; 0.55) ***	0.07	11	
Emergent Approaches	0.39 (0.29; 0.50) ***	0.05	25	
Members’ Leadership	0.40 (0.25; 0.56) ***	0.08	14	
**Leadership evaluation**				F(1,49) = 1.06, p = .31
Auto Leadership Evaluation	0.45 (0.31, 0.58) ***	0.07	18	
Hetero Leadership evaluation	0.37 (0.28; 0.45) ***	0.04	33	
**AP evaluation**				F(1, 48) = 0.13, p = .72
Auto AP evaluation	0.38 (0.28; 0.48) ***	0.06	32	
Hetero AP evaluation	0.34 (0.16; 0.52) ***	0.09	18	
**Type of AP**				F(1, 47) = 0.19, p = .67
Individual AP	0.40 (0.32; 0.49) ***	0.04	36	
Team AP	0.37 (0.21;0.52) ***	0.08	13	
**Organization Type**				F(1, 36) = 0.01, p = .95
Private	0.35 (0.23; 0.48) ***	0.06	22	
Public	0.25 (0.19; 0.50) ***	0.08	16	
**Company Job Sectors**				F(3, 42) = 1.02, p = .39
Healthcare and human services	0.34 (0.19; 0.49) ***	0.07	14	
Market services	0.45 (.32; 0.59) ***	0.07	15	
Manufactory industries	0.52 (0.26; 0.77) ***	0.13	3	
Mixed	0.32 (0.15; 0.48) ***	0.08	14	
**Organizational changes**				F(1, 48) = 0.87, p = .36
No	0.41 (0.33; 0.50) ***	0.04	38	
Yes	0.32 (0.15; 0.49)***	0.09	12	
**Research design**				F(1, 49) = 0.52, p = .48
Cross-sectional	0.38 (0.29; 0.46)	0.04	39	
Longitudinal	0.44 (0.28; 0.59)	0.08	12	
**Effect**				F(1, 50) = 4.20, p = .046
Beta (ß)	0.22 (0.03; 0.40)*	0.09	6	
Pearson (r)	0.41 (0.34; 0.49) ***	0.04	46	

*** p < .001.

Finally, our publication bias analysis did not yield any significant results for both reciprocal of sample size, b = -2.27, SE = 9.27, t(50) = -0.25, p = .81, and standard error of effect size, b = -0.17, SE = 1.40, t(50) = 0.12, p = 0.90, thus suggesting no evident publication bias.

## Meta-analysis discussion

Results indicated that, in line with our first hypothesis (H1), leadership had a positive effect on the promotion of adaptivity at work; it suggested that leadership supported the implementation of adaptive behaviors regardless of style chosen. Indeed, the direction of change in the organizational environment could be top-down, whenever it started from the management that provided guidelines on aims and methods of implementation; or it could be bottom-up whenever the change proposal came from the employees’ proactive process. Based on our meta-analysis results, it was possible to assume that leadership was functional to the achievement of adaptivity, whether it was exercised by top management or by the employees themselves (like shared and self-leadership styles).

Accordingly, Schmitt and colleagues [149] already stressed that motivation and personal initiative and activation are very important in adaptive performance and are aspects in which leadership can play an important role. Leadership helps employees not only to perform better in their tasks but also helps collaborators to get involved, to go beyond the prescribed tasks, to be responsible for the outcomes of the activity, as well as it encourages team members to express ideas and suggestions when adapting to changing organizational circumstances.

However, contrary to expectations, there was no evidence supporting the existence of a stronger relationship between one or more leadership styles and AP (H2) and there was no difference between more or less top-down styles (H3). This could be due to the different contexts in which studies had been conducted and the nature of work. Furthermore, the typology of organizational changes, in terms of structural, technological, emergent and cultural change, that employees faced can vary greatly, imposing very different styles and adaptivity behaviors.

Even though no differences had emerged from the leadership groups included in our research (neo-charismatic, emergent and members’ leadership) and the company sectors, as was expected from the systematic review, leadership self-evaluation tended to be more correlated with AP compared to hetero-evaluation. This could be due to the influence of social desirability and self-serving bias in self-assessing, which can induce someone to present a more favourable leadership style, as opposed to when it is assessed by others [150–152] or the style assessed through self-evaluation. Particularly, especially in shared and self-leadership, employees assess their own behavior monitoring and control, which personally involves them and their group. It is possible that these processes are the precursors to the activation of a proactive behavior which lead to achieve organizational goals.

## Conclusions and future directions

In conclusion, while work adaptivity in the past primarily referred to prescribed role behaviors specified in job descriptions, today AP encompasses aspects of creativity, versatility, and stress management. The interdependence of organizational roles and the emphasis on teamwork have transformed the concept of adaptation, evolving it from solely an individual process to a collective form of performance [153]. The ability to manage emotions that arise during unpredictable situations and the ability to maintain an open channel of communication prove that leadership can support change [154].

It would seem more central, regardless of style, to the real involvement of the leader in a process of exchange, communication, and interdependency with collaborators [154,155].

These findings contribute to the literature on the association between leadership and AP as they a) provided a summary of the effect size and variability of this association, and b) discovered a high variance of this association, which was not previously evidenced in literature. Hence, although none of the considered variables emerged as significant mediators, present findings clarify the extent to which leadership and AP are associated. So, it could become a starting point to deepen the knowledge about organizational variables and leadership-related variables acting as predictive of AP.

Future research should integrate a comprehensive longitudinal study design to explore the interplay between leadership styles, AP, and organizational culture. This study would quantify how leadership styles are influenced by organizational norms, rules, assumptions, beliefs, and values and how these factors promote or inhibit AP, compared with task and contextual performance [155]. Validated scales for leadership styles, a developed AP measurement, and organizational culture assessment tools should be employed. Furthermore, the attention paid to *ethicability*, as related to sustainable job performance, is growing and HR management practices are more attentive to employees’ wellbeing and development, being centered on human capital as one of the factors that contribute to a company’s growth. Future studies could investigate a type of AP that is sustainable over time and understand human limits, especially those connected with how to use and maintain psychological, personal and organizational resources that can ensure long-term sustainable AP [156]. Sustainability could be a new coordinate through which we can read the role of leadership in adaptive performance.

### Study limitations

In order to generalize the findings, it will be necessary to expand with gray literature so to have additional leadership styles to include in the research and to compare. Furthermore, the heterogeneity of the studies compared and the intrinsic limitations of the measurement instruments used in the literature that was reviewed should be considered, in particular the differences between scales used in the studies on self or hetero- assessment. The review was not registered and a protocol review was not prepared.

### Practical implications

The practical implications derived from the meta-analysis emphasize the critical role of leadership in enhancing AP within organizations. Given the absence of a one-size-fits-all leadership style for improving AP, organizations might benefit from adopting flexible leadership approaches. This adaptability allows leadership behaviors to be tailored to the team’s needs and the specific context of organizational changes.

In summary, the study advocates for leadership practices that support adaptability through skill development, flexibility, self-awareness, and alignment with organizational objectives, aiming to create more resilient and adaptable organizations. Additionally, findings suggest investing in organizational training aimed to increase awareness about the importance of governance and managerial roles in supporting changes and their influence on subordinates’ adaptivity. The awareness that leadership creates the basis to foster adaptivity should stimulate managers to have an active role in preparing and supporting collaborators in organizational development paths, facing resistance to change which is often the result of a lack of sharing and participation in which leadership has a crucial influence.

## Supporting information

S1 Appendix PRISMA checklist. (PDF)
