# Supplementary material for: The relationship between leadership and adaptive performance: A systematic review and meta-analysis
Source: PLoS One. 2024 Oct 18;19(10):e0304720. doi: 10.1371/journal.pone.0304720 (PMC11488715; doi:10.1371/journal.pone.0304720)
Supplement: Peer review history

# Peer review: Decision Letter 0

**DOI:** 10.1371/journal.pone.0304720.r001

**Authors:** Faisal Shafique Butt

16 Apr 2024

PONE-D-24-08921The relationship between leadership and adaptive performance: A systematic review and meta-analysisPLOS ONE

Dear Dr. Bonini,

Thank you for submitting your manuscript to PLOS ONE. After careful consideration, we feel that it has merit but does not fully meet PLOS ONE’s publication criteria as it currently stands. Therefore, we invite you to submit a revised version of the manuscript that addresses the points raised during the review process.

Please submit your revised manuscript by May 31 2024 11:59PM. If you will need more time than this to complete your revisions, please reply to this message or contact the journal office at plosone@plos.org. When you're ready to submit your revision, log on to https://www.editorialmanager.com/pone/ and select the 'Submissions Needing Revision' folder to locate your manuscript file.

Please include the following items when submitting your revised manuscript:

- A rebuttal letter that responds to each point raised by the academic editor and reviewer(s). You should upload this letter as a separate file labeled 'Response to Reviewers'.
- A marked-up copy of your manuscript that highlights changes made to the original version. You should upload this as a separate file labeled 'Revised Manuscript with Track Changes'.
- An unmarked version of your revised paper without tracked changes. You should upload this as a separate file labeled 'Manuscript'.

If you would like to make changes to your financial disclosure, please include your updated statement in your cover letter. Guidelines for resubmitting your figure files are available below the reviewer comments at the end of this letter.

If applicable, we recommend that you deposit your laboratory protocols in protocols.io to enhance the reproducibility of your results. Protocols.io assigns your protocol its own identifier (DOI) so that it can be cited independently in the future. For instructions see: https://journals.plos.org/plosone/s/submission-guidelines#loc-laboratory-protocols. Additionally, PLOS ONE offers an option for publishing peer-reviewed Lab Protocol articles, which describe protocols hosted on protocols.io. Read more information on sharing protocols at https://plos.org/protocols?utm_medium=editorial-email&utm_source=authorletters&utm_campaign=protocols.

We look forward to receiving your revised manuscript.

Kind regards,

Faisal Shafique Butt, Ph.D

Academic Editor

PLOS ONE

Journal Requirements:

When submitting your revision, we need you to address these additional requirements.

1. Please ensure that your manuscript meets PLOS ONE's style requirements, including those for file naming. The PLOS ONE style templates can be found at 

https://journals.plos.org/plosone/s/file?id=wjVg/PLOSOne_formatting_sample_main_body.pdf and 

https://journals.plos.org/plosone/s/file?id=ba62/PLOSOne_formatting_sample_title_authors_affiliations.pdf

2. In the online submission form, you indicated that [The datasets are available from the corresponding author on reasonable request.]. 

All PLOS journals now require all data underlying the findings described in their manuscript to be freely available to other researchers, either 1. In a public repository, 2. Within the manuscript itself, or 3. Uploaded as supplementary information.

This policy applies to all data except where public deposition would breach compliance with the protocol approved by your research ethics board. If your data cannot be made publicly available for ethical or legal reasons (e.g., public availability would compromise patient privacy), please explain your reasons on resubmission and your exemption request will be escalated for approval. 

3. When completing the data availability statement of the submission form, you indicated that you will make your data available on acceptance. We strongly recommend all authors decide on a data sharing plan before acceptance, as the process can be lengthy and hold up publication timelines. Please note that, though access restrictions are acceptable now, your entire data will need to be made freely accessible if your manuscript is accepted for publication. This policy applies to all data except where public deposition would breach compliance with the protocol approved by your research ethics board. If you are unable to adhere to our open data policy, please kindly revise your statement to explain your reasoning and we will seek the editor's input on an exemption. Please be assured that, once you have provided your new statement, the assessment of your exemption will not hold up the peer review process.

4. Please review your reference list to ensure that it is complete and correct. If you have cited papers that have been retracted, please include the rationale for doing so in the manuscript text, or remove these references and replace them with relevant current references. Any changes to the reference list should be mentioned in the rebuttal letter that accompanies your revised manuscript. If you need to cite a retracted article, indicate the article’s retracted status in the References list and also include a citation and full reference for the retraction notice.

Additional Editor Comments:

Please do the changes as suggested by the reviewers

[Note: HTML markup is below. Please do not edit.]

Reviewers' comments:

Reviewer's Responses to Questions

**Comments to the Author**

1. Is the manuscript technically sound, and do the data support the conclusions?

The manuscript must describe a technically sound piece of scientific research with data that supports the conclusions. Experiments must have been conducted rigorously, with appropriate controls, replication, and sample sizes. The conclusions must be drawn appropriately based on the data presented. 

Reviewer #1: Partly

Reviewer #2: Yes

**********

2. Has the statistical analysis been performed appropriately and rigorously? 

Reviewer #1: No

Reviewer #2: Yes

**********

3. Have the authors made all data underlying the findings in their manuscript fully available?

The PLOS Data policy requires authors to make all data underlying the findings described in their manuscript fully available without restriction, with rare exception (please refer to the Data Availability Statement in the manuscript PDF file). The data should be provided as part of the manuscript or its supporting information, or deposited to a public repository. For example, in addition to summary statistics, the data points behind means, medians and variance measures should be available. If there are restrictions on publicly sharing data—e.g. participant privacy or use of data from a third party—those must be specified.

Reviewer #1: Yes

Reviewer #2: Yes

**********

4. Is the manuscript presented in an intelligible fashion and written in standard English?

PLOS ONE does not copyedit accepted manuscripts, so the language in submitted articles must be clear, correct, and unambiguous. Any typographical or grammatical errors should be corrected at revision, so please note any specific errors here.

Reviewer #1: Yes

Reviewer #2: Yes

**********

5. Review Comments to the Author

Please use the space provided to explain your answers to the questions above. You may also include additional comments for the author, including concerns about dual publication, research ethics, or publication ethics. (Please upload your review as an attachment if it exceeds 20,000 characters)

Reviewer #1: Dear Authors,

I have gone through the article and my comments to improve the manuscript are as under

1. What is the contribution of your study in the existing set of literature, the study is silent on it, please clearly document the contribution of your study.

2. How your study is different from earlier studies, please clearly elaborate.

3. What is the research gap in the existing literature, the study is silent on it. Please elaborate.

4. Can this study construct a comprehensive scale based on this study for future research, if so, then please make a part of this paper.

5. The authors have used the word “thanks to” while recognizing the theorists. Such words are normally not used in the literature.

6. Table 1 and Table 2 should be shifted in the Appendix.

7. The data should be represented through Venn diagrams, histograms, etc. for better presentation.

8. What are the suggestions to policymakers as an outcome of research, without it, the research work has no meaning. The given policy implications are meager, please elaborate on this section keeping in mind the outcome of the study.

9. I suggest the Key Characteristics of Each of the Leadership styles in Tabular form by giving the brief characteristics for the reader’s interest.

10. The region-wise or country-wise studies should be shown in the Venn diagram for better understanding.

11. The study has not been given in the conceptual model of AP.

12. The study has designed the hypothesis in the LR part, but later on, there is no discussion on these hypotheses. Authors are requested to discuss the results with respect to these hypotheses. Besides, only two hypotheses are meager for the study, please consider further hypotheses for the study.

13. I suggest a brief section on the theories of leadership and adaptive performance.

Regards

Reviewer #2: Your abstract provides a clear overview of the purpose, methodology, and findings of your research. It effectively communicates the importance of studying the relationship between leadership styles and adaptive performance (AP) in organizational settings undergoing rapid changes. The inclusion of the theoretical reference framework and the methodology employed adds to the comprehensiveness of the abstract. However, I suggest rephrasing the sentence "The theoretical reference framework for the study of leadership and AP was outlined first" for clarity and conciseness.

While the introduction effectively highlights the importance of adaptive performance in the face of changing work environments, it could provide more context on the specific gaps in existing literature that the study aims to address.

While the introduction briefly discusses the theoretical underpinnings of adaptive performance and leadership, it would benefit from a more in-depth exploration of relevant theories and their application to the study.

The transition from discussing the importance of leadership to outlining the aims and objectives of the study could be smoother to improve the flow of the introduction. Furthermore, I would suggest to remove heading provide for aims and objective for more readability.

The introduction could benefit from a brief discussion on the significance and practical implications of the study's findings for organizations and leadership development strategies.

The literature background provided offers valuable insights into adaptive performance (AP) and leadership styles, however, it is better to consider some updated and current literature in literature section.

This review explores how different leadership styles influence adaptive performance in the workplace. Examining 34 articles from peer-reviewed journals, it sheds light on the impact of styles like transformational and servant leadership.

The review analyzes 34 articles on leadership styles and adaptive performance (AP) in the workplace. Findings confirm a significant positive relationship between leadership and AP across different industries. Transformational, transactional, and servant leadership styles are particularly influential. The meta-analysis supports these findings, considering various moderators. No publication bias is detected. Overall, the review underscores the importance of leadership in fostering adaptive behaviors among employees.

The meta-analysis found a significant and moderate correlation (r = .37) between leadership and adaptive performance (AP), indicating a robust relationship. Heterogeneity across studies was high, but most moderators did not show significant effects. However, a small but significant difference was found between effect types (ß vs r). No publication bias was detected. Overall, the analysis highlights the consistent influence of leadership on AP across different organizational contexts and evaluation methods.

While the discussion is well-articulated, it would benefit from more robust evidence supporting a stronger relationship between specific leadership styles and adaptivity at work. Moreover, the heavy reliance on self-assessment for leadership evaluation may introduce bias into the findings. To enhance the validity of the conclusions, it would be prudent to incorporate more supportive literature evidence and consider alternative methods of leadership assessment to mitigate potential biases. Also try to avoid abbreviation for more fluency and clarity.

**********

6. PLOS authors have the option to publish the peer review history of their article (what does this mean?). If published, this will include your full peer review and any attached files.

If you choose “no”, your identity will remain anonymous but your review may still be made public.

**Do you want your identity to be public for this peer review?** For information about this choice, including consent withdrawal, please see our Privacy Policy.

Reviewer #1: No

Reviewer #2: No

**********

[NOTE: If reviewer comments were submitted as an attachment file, they will be attached to this email and accessible via the submission site. Please log into your account, locate the manuscript record, and check for the action link "View Attachments". If this link does not appear, there are no attachment files.]

While revising your submission, please upload your figure files to the Preflight Analysis and Conversion Engine (PACE) digital diagnostic tool, https://pacev2.apexcovantage.com/. PACE helps ensure that figures meet PLOS requirements. To use PACE, you must first register as a user. Registration is free. Then, login and navigate to the UPLOAD tab, where you will find detailed instructions on how to use the tool. If you encounter any issues or have any questions when using PACE, please email PLOS at figures@plos.org. Please note that Supporting Information files do not need this step.

# Author comment: Author response to Decision Letter 0

**DOI:** 10.1371/journal.pone.0304720.r002

14 May 2024

Response to Reviewers

Journal Requirements:

When submitting your revision, we need you to address these additional requirements.

1. Please ensure that your manuscript meets PLOS ONE's style requirements, including those for file naming. The PLOS ONE style templates can be found at 

https://journals.plos.org/plosone/s/file?id=wjVg/PLOSOne_formatting_sample_main_body.pdf and 

https://journals.plos.org/plosone/s/file?id=ba62/PLOSOne_formatting_sample_title_authors_affiliations.pdf

By what is requested, Level 2 and 3 headings were modified using 16 and 14pt font

2. In the online submission form, you indicated that [The datasets are available from the corresponding author on reasonable request.]. All PLOS journals now require all data underlying the findings described in their manuscript to be freely available to other researchers, either 1. In a public repository, 2. Within the manuscript itself, or 3. Uploaded as supplementary information. This policy applies to all data except where public deposition would breach compliance with the protocol approved by your research ethics board. If your data cannot be made publicly available for ethical or legal reasons (e.g., public availability would compromise patient privacy), please explain your reasons on resubmission and your exemption request will be escalated for approval. 

Data for systematic review have been extrapolated from the articles cited in the text and are partially within the manuscript itself. The meta-analyzed database has been now added in OSF and made available at this link:

https://osf.io/rz82g/?view_only=8e62442ac7d3459cb75508f15fc41dbc

3. When completing the data availability statement of the submission form, you indicated that you will make your data available on acceptance. We strongly recommend all authors decide on a data sharing plan before acceptance, as the process can be lengthy and hold up publication timelines. Please note that, though access restrictions are acceptable now, your entire data will need to be made freely accessible if your manuscript is accepted for publication. This policy applies to all data except where public deposition would breach compliance with the protocol approved by your research ethics board. If you are unable to adhere to our open data policy, please kindly revise your statement to explain your reasoning and we will seek the editor's input on an exemption. Please be assured that, once you have provided your new statement, the assessment of your exemption will not hold up the peer review process.

Please see the comment above.

4. Please review your reference list to ensure that it is complete and correct. If you have cited papers that have been retracted, please include the rationale for doing so in the manuscript text, or remove these references and replace them with relevant current references. Any changes to the reference list should be mentioned in the rebuttal letter that accompanies your revised manuscript. If you need to cite a retracted article, indicate the article’s retracted status in the References list and also include a citation and full reference for the retraction notice.

As you suggested, we reviewed the reference list. Ten references were added: 

33. Desti Febrian, W., Muliyati, Lily, Rajab, M. ., & Thamrin AR, M. . (2023). Transactional Leadership: Employee Performance and Organizational Performance (Literature Review). East Asian Journal of Multidisciplinary Research, 2(3), 1129–1142. https://doi.org/10.55927/eajmr.v2i3.3410

35. Hall, M. L., Meyer, C. K., & Clapham, M. M. (2023). Leadership theories and styles understood and synthesised. Journal of Business and Behavioral Sciences, 35, 93–102.

42. Martinez, S.-A. and Leija, N. "Distinguishing Servant Leadership from Transactional and Transformational Leadership", Advances in Developing Human Resources, 25(3), pp. 141-188. Copyright © 2023, The Author(s) . Reprinted by permission of SAGE Publications. https://doi.org/10.1177/15234223231175845

46. Yagil, D., & Oren, R. (2021). Servant leadership, engagement, and employee outcomes: The moderating roles of proactivity and job autonomy. Revista de Psicología del Trabajo y de las Organizaciones, 37(1), 58-65. 10.5093/jwop2021a1

50. Ahmed, E. I. (2023). A systematic review of authentic leadership literature in educational research from 1997 to 2021. Educational Management Administration & Leadership, 17411432221120467.

psychological empowerment. Management Research Review, 42(5), 605-624.

56. Kim, M., Beehr, T. A., & Prewett, M. S. (2018). Employee Responses to Empowering Leadership: A Meta-Analysis. Journal of Leadership & Organizational Studies, 25(3), 257–276. https://doi.org/10.1177/1548051817750538

57. Kundu, S. C., Kumar, S., & Gahlawat, N. (2019). Empowering leadership and job performance: mediating role of 

113. Rus D, Van Knippenberg D, Wisse B. Leader self-definition and leader self-serving behavior. Leadersh Q. 2010 Jun;21(3):509–29. 

114. Lammers J, Burgmer P. Power increases the self‐serving bias in the attribution of collective successes and failures. Eur J Soc Psychol. 2019 Aug;49(5):1087–95. 

115. Dobbins GH, Russell JM. Self-Serving Biases in Leadership: A Laboratory Experiment. J Manag. 1986 Dec;12(4):475–83. 

Reviewers' comments:

Reviewer's Responses to Questions

Comments to the Author

1. Is the manuscript technically sound, and do the data support the conclusions?

The manuscript must describe a technically sound piece of scientific research with data that supports the conclusions. Experiments must have been conducted rigorously, with appropriate controls, replication, and sample sizes. The conclusions must be drawn appropriately based on the data presented. 

Reviewer #1: Partly

Reviewer #2: Yes

2. Has the statistical analysis been performed appropriately and rigorously? 

Reviewer #1: No

Reviewer #2: Yes

3. Have the authors made all data underlying the findings in their manuscript fully available?

The PLOS Data policy requires authors to make all data underlying the findings described in their manuscript fully available without restriction, with rare exception (please refer to the Data Availability Statement in the manuscript PDF file). The data should be provided as part of the manuscript or its supporting information, or deposited to a public repository. For example, in addition to summary statistics, the data points behind means, medians and variance measures should be available. If there are restrictions on publicly sharing data—e.g. participant privacy or use of data from a third party—those must be specified. 

Reviewer #1: Yes

Reviewer #2: Yes

4. Is the manuscript presented in an intelligible fashion and written in standard English?

PLOS ONE does not copyedit accepted manuscripts, so the language in submitted articles must be clear, correct, and unambiguous. Any typographical or grammatical errors should be corrected at revision, so please note any specific errors here.

Reviewer #1: Yes

Reviewer #2: Yes

5. Review Comments to the Author

Please use the space provided to explain your answers to the questions above. You may also include additional comments for the author, including concerns about dual publication, research ethics, or publication ethics. (Please upload your review as an attachment if it exceeds 20,000 characters)

Reviewer #1: Dear Authors,

I have gone through the article and my comments to improve the manuscript are as under

1. What is the contribution of your study in the existing set of literature, the study is silent on it, please clearly document the contribution of your study. 

2. How your study is different from earlier studies, please clearly elaborate.

1.2. As you suggested, we have added a mention of the contribution of this study and the difference from earlier ones in the “conclusion” paragraph (lines 567 – 572), which can enrich the paper. This is the phrase we inserted: “These findings contribute to the literature on the association between leadership and AP as they a) provided a summary of the effect size and variability of this association, and b) discovered a high variance of this association, which was not previously evidenced in the literature. Hence, although none of the considered variables emerged as significant mediators, present findings clarify the extent to which leadership and AP are associated. So, it could become a starting point to deepen the knowledge about organizational variables and leadership-related variables acting as predictive of AP.”

3. What is the research gap in the existing literature, the study is silent on it. Please elaborate.

3. Thank you for this advice. We mentioned the research gap in the “introduction” paragraph. Now we added this sentence: “Despite the recognized importance of leadership in facilitating the adaptive performance, the understanding of how different leadership styles specifically contribute to this dynamic remains fragmented in literature” (lines 59-61.)

4. Can this study construct a comprehensive scale based on this study for future research, if so, then please make a part of this paper.

4. Thank you for your suggestion. However, could you give us a clarification about your request? Regarding future research, we suggest integrating a comprehensive longitudinal study design to explore the interplay between leadership styles, AP, and organizational culture (see lines 573-578). Regarding the development of a scale based on this study, we believe that this study does not allow us to construct a comprehensive scale for future research. Still, we appreciate your suggestion about this point. 

5. The authors have used the word “thanks to” while recognizing the theorists. Such words are normally not used in the literature.

5. As you pointed out, we removed the words “thanks to” and changed it to “because of” or replaced it with better wording.

6. Table 1 and Table 2 should be shifted in the Appendix.

6. We understand the reviewer’s concern and acknowledge that shifting the tables to the appendix would make the text easier to follow. However, unless the Editor raises space concerns, we would like to maintain tables in the text, as we believe that this would facilitate readers’ understanding.

7. The data should be represented through Venn diagrams, histograms, etc. for better presentation.

7. Could you please clarify what kind of data should be better represented in Venn diagrams or histograms? We tried to cross different types of data (country, leadership style, job sector, and auto and hetero leadership evaluation) but the results were not significant either at a content level or at a visual level. For more clarity, we inserted in Table 2 the number of articles selected for review and meta-analysis, beside the “leadership style” we added several items using it.

8. What are the suggestions to policymakers as an outcome of research, without it, the research work has no meaning. The given policy implications are meager, please elaborate on this section keeping in mind the outcome of the study. 

8. Thank you for this comment which allows us to highlight the practical implications that can also be used from an organizational development perspective.

Concerning this point, there was a sentence in the “Practical implications” paragraph: “In summary, the study advocates for leadership practices that support adaptability through skill development, flexibility, self-awareness, and alignment with organizational objectives, aiming to create more resilient and adaptable organizations. 

Now we also have added: “Additionally, findings suggest investing in organizational training aimed to increase awareness about the importance of governance and managerial roles in supporting changes and their influence on subordinates’ adaptivity. The awareness that leadership creates the basis to foster adaptivity should stimulate managers to have an active role in preparing and supporting collaborators in organizational development paths, facing resistance to change which is often the result of a lack of sharing and participation in which leadership has a crucial influence.” (lines 600-606).

9. I suggest the Key Characteristics of Each of the Leadership styles in Tabular form by giving the brief characteristics for the reader’s interest.

9. Following your suggestion, we added a table to resume the principal features of different leadership styles (page 9 - line 225).

10. The region-wise or country-wise studies should be shown in the Venn diagram for better understanding.

10. It is difficult for us to understand how to use the Venn diagram to illustrate the distribution of countries. We will be available to add this diagram if we receive from you further guidance. 

Alternatively, do you think it would be useful to include a histogram of the countries? 

Regarding the country of origin of the studies, you can find this information in the text (lines 323-324). In the previous version, the number of Asian articles was wrong, we modified it in “Asia (n. 17)”. 

11. The study has not been given in the conceptual model of AP.

11. As you suggested, the conceptual model of AP was further explored in the literature background paragraph (lines 101 - 110) trying to illustrate and deepen the differences and similarities between the dimensions of the different theoretical models (Pulakos’ and Neal, Griffin &Parker’s model). 

12. The study has designed the hypothesis in the LR part, but later on, there is no discussion on these hypotheses. Authors are requested to discuss the results with respect to these hypotheses. Besides, only two hypotheses are meager for the study, please consider further hypotheses for the study. 

12. Following your suggestion, we enlarged the hypotheses and reported the hypotheses in the “Meta-analysis discussion” paragraph.

13. I suggest a brief section on the theories of leadership and adaptive performance. 

13. In the literature background paragraph, we describe AP definitions and leadership theories. 

Thank you for your suggestion: at the end of the paragraph, we added a table to resume the principal features of different leadership styles.

Regards

Reviewer #2: Your abstract provides a clear overview of the purpose, methodology, and findings of your research. It effectively communicates the importance of studying the relationship between leadership styles and adaptive performance (AP) in organizational settings undergoing rapid changes. The inclusion of the theoretical reference framework and the methodology employed adds to the comprehensiveness of the abstract. However, I suggest rephrasing the sentence "The theoretical reference framework for the study of leadership and AP was outlined first" for clarity and conciseness. 

1. Thank you very much for your precious suggestions that allow us to improve and enhance our paper. We rephrased the sentence as you indicated. 

While the introduction effectively highlights the importance of adaptive performance in the face of changing work environments, it could provide more context on the specific gaps in existing literature that the study aims to address 

2. We have already mentioned the research gap in the “introduction” paragraph but, following your suggestion, we added a sentence read as follows: “Despite the recognized importance of leadership in facilitating adaptive performance, the understanding of how different leadership styles specifically contribute to this dynamic remain fragmented in literature” (lines 59-61)

While the introduction briefly discusses the theoretical underpinnings of adaptive performance and leadership, it would benefit from a more in-depth exploration of relevant theories and their application to the study. 

3. Following your precious advice, the conceptual model of AP was further explored in the literature background paragraph (from line 101 to 118) trying to illustrate and deepen the differences and similarities between the dimensions of the different theoretical models (Pulakos and Neal, Griffin &Parker). Also, in the paragraph “Leadership styles: literature overview and the relationship with job adaptive performance”, we choose to discuss the more relevant leadership theories and their relationship with AP for the past literature. For more cl

Attachment Submitted filename: Response to Reviewers.docx

# Editor report: Decision Letter 1

**DOI:** 10.1371/journal.pone.0304720.r003

**Authors:** Faisal Shafique Butt

17 May 2024

The relationship between leadership and adaptive performance: A systematic review and meta-analysis

PONE-D-24-08921R1

Dear Dr. Bonini,

We’re pleased to inform you that your manuscript has been judged scientifically suitable for publication and will be formally accepted for publication once it meets all outstanding technical requirements.

Within one week, you’ll receive an e-mail detailing the required amendments. When these have been addressed, you’ll receive a formal acceptance letter and your manuscript will be scheduled for publication.

An invoice will be generated when your article is formally accepted. Please note, if your institution has a publishing partnership with PLOS and your article meets the relevant criteria, all or part of your publication costs will be covered. Please make sure your user information is up-to-date by logging into Editorial Manager at Editorial Manager® and clicking the ‘Update My Information' link at the top of the page. If you have any questions relating to publication charges, please contact our Author Billing department directly at authorbilling@plos.org.

If your institution or institutions have a press office, please notify them about your upcoming paper to help maximize its impact. If they’ll be preparing press materials, please inform our press team as soon as possible -- no later than 48 hours after receiving the formal acceptance. Your manuscript will remain under strict press embargo until 2 pm Eastern Time on the date of publication. For more information, please contact onepress@plos.org.

Kind regards,

Faisal Shafique Butt, Ph.D

Academic Editor

PLOS ONE

Additional Editor Comments (optional):

Reviewers' comments:

# Editor report: Acceptance letter

**DOI:** 10.1371/journal.pone.0304720.r004

**Authors:** Faisal Shafique Butt

30 Jul 2024

PONE-D-24-08921R1 

PLOS ONE

Dear Dr. Bonini, 

I'm pleased to inform you that your manuscript has been deemed suitable for publication in PLOS ONE. Congratulations! Your manuscript is now being handed over to our production team.

At this stage, our production department will prepare your paper for publication. This includes ensuring the following:

* All references, tables, and figures are properly cited

* All relevant supporting information is included in the manuscript submission,

* There are no issues that prevent the paper from being properly typeset

If revisions are needed, the production department will contact you directly to resolve them. If no revisions are needed, you will receive an email when the publication date has been set. At this time, we do not offer pre-publication proofs to authors during production of the accepted work. Please keep in mind that we are working through a large volume of accepted articles, so please give us a few weeks to review your paper and let you know the next and final steps. 

Lastly, if your institution or institutions have a press office, please let them know about your upcoming paper now to help maximize its impact. If they'll be preparing press materials, please inform our press team within the next 48 hours. Your manuscript will remain under strict press embargo until 2 pm Eastern Time on the date of publication. For more information, please contact onepress@plos.org.

If we can help with anything else, please email us at customercare@plos.org.

Thank you for submitting your work to PLOS ONE and supporting open access. 

Kind regards, 

PLOS ONE Editorial Office Staff

on behalf of

Dr. Faisal Shafique Butt 

Academic Editor

PLOS ONE
